# The genome of *Vitis vinifera* cv. Mgaloblishvili reveals resistance and susceptibility factors to downy mildew in the *Rpv29* and *Rpv31* loci

**DOI:** 10.1093/hr/uhaf055

**Published:** 2025-02-20

**Authors:** Valentina Ricciardi, Andrea Minio, Melanie Massonnet, Alexander H J Wittenberg, Rosa Figueroa-Balderas, David Maghradze, Silvia Laura Toffolatti, Osvaldo Failla, Dario Cantu, Gabriella De Lorenzis

**Affiliations:** Department of Agricultural and Environmental Sciences, University of Milan, 2 G. Celoria 2 str., 20133, Milan, Italy; Institute of Biosciences and Bioresources, National Research Council, 153 Ugo La Malfa str., 90146, Palermo, Italy; Institute for Biomedicine, Eurac Research, 21 A. Volta Straße str., 39100, Bozen, Italy; Department of Viticulture and Enology, University of California Davis, 595 Hilgard Lane, 95616, Davis, CA, USA; Department of Viticulture and Enology, University of California Davis, 595 Hilgard Lane, 95616, Davis, CA, USA; KeyGene, Agro Business Park 90, 6708 PW, Wageningen, The Netherlands; Department of Viticulture and Enology, University of California Davis, 595 Hilgard Lane, 95616, Davis, CA, USA; Faculty of Viticulture and Winemaking, Caucasus International University, 73 Chargali str., 0159, Tbilisi, Georgia; Faculty of Agrarian Sciences and BiosystemsEngineering, Georgian Technical University, 77 Kostava str., 0171, Tbilisi, Georgia; Department of Agricultural and Environmental Sciences, University of Milan, 2 G. Celoria 2 str., 20133, Milan, Italy; Department of Agricultural and Environmental Sciences, University of Milan, 2 G. Celoria 2 str., 20133, Milan, Italy; Department of Viticulture and Enology, University of California Davis, 595 Hilgard Lane, 95616, Davis, CA, USA; Genome Center, University of California Davis, 451 Health Sciences Dr., 95616, Davis, CA, USA; Department of Agricultural and Environmental Sciences, University of Milan, 2 G. Celoria 2 str., 20133, Milan, Italy

## Abstract

Mgaloblishvili, a grapevine variety from Georgia (Southern Caucasus), exhibits a unique resistance mechanism against downy mildew. Mgaloblishvili resistance mechanism, involving pathogen recognition, activation of ethylene signalling pathway, and structural and chemical defences, is mediated by the resistance loci *Rpv29*, *Rpv30*, and *Rpv31*. Mgaloblishvili genome was sequenced using PacBio HiFi, resulting in a chromosome-scale diploid assembly of 986 Mbp, including 58 912 predicted protein-coding genes across two phased chromosome sets. Comparative analysis with the susceptible PN40024 genome allowed us to identify differences in structure, gene content, and gene expression, as well as the impact of structural variants (SVs) and single nucleotide polymorphisms (SNPs) between Mgaloblishvili and PN40024 loci. Resistance haplotypes were identified through DNA sequencing of a self-pollinated Mgaloblishvili population. Compared to orthologous regions in PN40024, the *Rpv29* locus in Mgaloblishvili exhibits reduced gene content, while the *Rpv31* locus has similar gene content. In both Mgaloblishvili and PN40024, most genes within these loci are associated with plant defence pathways. While genes in both genotypes perform similar functions, SVs and SNPs were identified as key determinants of the structural differences between the genomes. Defining the *Rpv30* locus was challenging due to ambiguous marker localization. DNA sequencing allowed us to identify resistance haplotypes for both *Rpv30* and *Rpv31* on Mgaloblishvili haplotype 2, though insights into the *Rpv29* locus remain limited. Our results indicate that Mgaloblishvili’s resistance is driven by numerous small SVs and SNPs, which lead to the loss of susceptibility factors and unique transcriptional regulation of defence-related genes.

## Introduction

Grapevine (*Vitis vinifera* L.) is one of the most important fruit crops worldwide (https://www.oiv.int/). One of the most detrimental diseases affecting grapevine production is downy mildew. The disease is caused by the oomycete *Plasmopara viticola* [(Berk. & Curt.) Berl. & de Toni], a biotrophic pathogen capable of infecting all the green tissues of grapevines, including stems and bunches [1]. This results in significant losses in both quantity and quality of grapes produced [2]. The disease is primarily managed through regular fungicide applications, a solution that is increasingly deemed unsustainable, particularly in light of current European regulations on fungicide registration and use (EU Regulation 1107/2009; Directive 2009/128/CE), and the growing concerns regarding fungicide resistance [3]. Adopting disease-resistant cultivars could be a key part of the solution. Breeding programmes have been focusing on introducing resistance loci from American and Asian *Vitis* wild species into *V. vinifera* elite cultivars [4]. However, this approach present drawbacks, due to issues related to the linkage drag of undesirable traits [5].

In recent years, some studies have highlighted a new and interesting source of resistant germplasm, the *V. vinifera* varieties from Georgia (South Caucasus) [6, 7]. The uniqueness of Georgian germplasm lies in its origin, which is considered to be one of the grapevine domestication centres [8, 9]. Among these interesting varieties, some cultivars, such as Mgaloblishvili, have been shown to possess an effective defence mechanism that limits *P. viticola* growth and sporulation [6, 7, 10]. This mechanism is associated with the overexpression of genes related to pathogen recognition, the ethylene signalling pathway, and the synthesis of antimicrobial compounds (VOCs) and enzymes, coupled with the deposition of structural barriers [10]. In comparison to Pinot noir, a susceptible cultivar, Mgaloblishvili showed a faster response against the pathogen, regulating the resistance genes within the first 24 h after inoculation [11]. Furthermore, Mgaloblishvili allowed the discovery of a new candidate gene of susceptibility to downy mildew, *VviLBDIf7*, which was downregulated during infection [12]. A transient RNA interference approach using exogenously applied dsRNA effectively downregulated the gene and reduced downy mildew infection [13].

A Genome-Wide Association Study (GWAS) was conducted to determine the genetic basis of Mgaloblishvili [14]. Three new loci of resistance to *P. viticola* were identified: (i) *Rpv29* on chromosome 14; (ii) *Rpv30* on chromosome 03; and (iii) *Rpv31* on chromosome 16. At the time, the only available chromosome-scale reference was the genome PN40024 12X.v2 [11]. Consequently, the genome was employed for the study. However, given its susceptibility to downy mildew, similar to the majority of *V. vinifera* cultivars, it lacked the adequate detail to investigate the genes involved in Mgaloblishvili resistance.

Working with grape genomes presents significant challenges, primarily due to grapevine cultivars being first-generation crosses from genetically distant parents. This issue, coupled with the accumulation of somatic mutations throughout the domestication history of grapevines, often results in highly heterozygous cultivars [12]. However, recent advancements in third-generation sequencing technologies, such as those offered by the Pacific Biosciences (PacBio) platform, have changed the landscape [15]. The introduction of long-read sequencing technologies, coupled with the development of new tools capable of effectively processing them, such as Falcon Unzip [16] or Hifiasm [17], has finally made it possible to assemble grapevine diploid genomes with a higher level of contiguity [18, 19].

In this study, we leveraged these technologies to assemble a chromosome-scale diploid genome for the resistant cultivar Mgaloblishvili. To the best of our knowledge, this is the first described genome of a *V. vinifera* accession resistant to downy mildew. By integrating genomic data from Mgaloblishvili with findings from a study based on the reference PN40024, we dissected the *Rpv29* and *Rpv31* resistance loci, offering a detailed analysis of their genetic content. Expression analysis of genes within each locus utilized previously published RNA sequencing data [10]. Additionally, DNA sequencing data from selected self-pollinated progeny of Mgaloblishvili enabled the identification of resistance haplotypes for *Rpv3*0 and *Rpv31*.

## Results

### Genome sequencing, assembly, and annotation

The genome of Mgaloblishvili was sequenced with PacBio HiFi. In total, 32.5 Gb of HiFi data were generated, representing a 60X coverage of haploid genome. The high data quality and sequencing depth led to the assembly of a chromosome-scaled diploid genome. The assembled sequences consist of two phased sets of 19 pseudochromosomes, ranging from 19.7 to 37.9 Mb (Table S1) with only 1.12% of the assembled bases (11.0 out of 985.6 Mb) remaining unplaced. The two alternative pseudomolecule sets show very similar sizes: 491.4 Mb for the first haplotype and 483.3 Mb for the second one, with 29 235 and 29 139 protein-coding genes, respectively (Table 1). The assembly completeness was assessed using Benchmarking Universal Single-Copy Orthologs (BUSCO) analysis and by mapping the annotated genes of the closely related *V. vinifera* PN40024. Each haplotype showed 99.0% of the complete core plant genes and 99.1% of PN40024 genes, confirming the completeness and the high quality of the assembly (Table 1).

**Table 1:** Summary of qualitative parameters of Mgaloblishvili genome. vMgal_v1.0 = diploid genome; vMgal_v1.0 hap1 = Mgaloblishvili haplotype 1; vMgal_v1.0 hap2 = Mgaloblishvili haplotype 2; vMgal_v1.0 unplaced = unplaced sequences (sequences that were not possible to assign to the belonging haplotype)

**Diploid genome**	**vMgal_v1.0**	**vMgal_v1.0** **hap1**	**vMgal_v1.0** **hap2**	**vMgal_v1.0** **unplaced**
**Cumulative length (Mb)**	985.6	491.4	483.3	11.0
**Number of sequences**	168	19	19	130
**Average sequence length (Mb)**	5.87	25.9	25.4	0.08
**Minimum sequence length (Mb)**	0.01	19.7	20.0	0.01
**Maximum sequence length (Mb)**	37.9	37.9	37.0	0.80
**N50 Length (Mb)**	25.8	25.9	25.2	0.27
**BUSCO (v 5.1.1) Eudicots_odb10**		98.2%	98.1%	0.8%
**BUSCO (v 5.1.1) Embryophytes_odb10**		98.1%	98.2%	0.9%
**BUSCO (v5.1.1) Viridiplantae_odb10**		99.2%	99.0%	1.1%
**Number of protein coding loci**		29 235	29 139	
**Number of CDSs**		43 363	43 121	628
**% of PN40024 mapping genes**		99.1%	99.1%	

### Identification of *Rpv29*, *Rpv30*, and *Rpv31* in Mgaloblishvili genome

**Figure 1:**
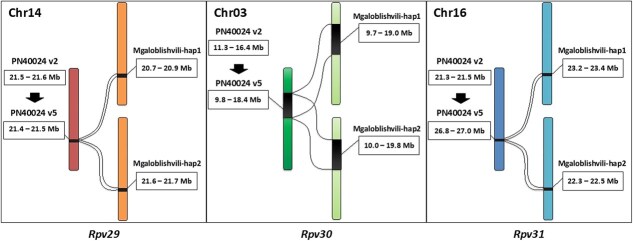
*Rpv29*, *Rpv30*, and *Rpv31* resistance loci position on the different chromosomes (Chr) of Mgaloblishvili genome. Per each locus, a comparison of the region between PN40024 v2, PN40024 v5, and Mgaloblishvili genome haplotypes (hap) is shown. Per each region the loci coordinates are indicated in Megabase (Mb). *Rpv29*: PN40024 v2 (133 573 bp), PN40024 v5 (132 688 bp), Mgaloblishvili haplotype 1 (139 026 bp), Mgaloblishvili haplotype 2 (129 470 bp); *Rpv30*: PN40024 v2 (5 082 810 bp), PN40024 v5 (8 542 018 bp), Mgaloblishvili haplotype 1 (9 234 319 bp), Mgaloblishvili haplotype 2 (9 820 061 bp); *Rpv31*: PN40024 v2 (193 814 bp), PN40024 v5 (194 502 bp), Mgaloblishvili haplotype 1 (206 678 bp), Mgaloblishvili haplotype 2 (199 473 bp).

*Rpv29*, *Rpv30*, and *Rpv31* resistance loci were identified on chromosomes 14, 03, and 16, respectively, through GWAS analysis [14], based on genotyping data generated using the *Vitis*18kSNP genotyping array [20]. The array probes were designed on the PN40024 12X v2 version of the genome. To assess the transferability of locus-location information to the Mgaloblishvili genome, the array SNP probes were mapped first to the PN40024 v4 and PN40024 v5 genome [21] and then to Mgaloblishvili haplotypes. The probes proved to be fully specific for *Rpv29* and *Rpv31*, which allowed to locate the position of both loci on both haplotypes of the Mgaloblishvili genome. Issues arose with *Rpv30*, as the probes for this locus mapped multiple times, likely due to repetitive sequences (Table S2). Aligning loci whole genomic regions between the PN40024 v5 and Mgaloblishvili genome assemblies clarified the *Vitis*18KSNP genotyping array data (Table S3), revealing the presence of a novel region in Mgaloblishvili in correspondence of the *Rpv30* locus. The *Rpv30* SNP probe showed the same ambiguity in the Mgaloblishvili genome, particularly in the case of haplotype 2. Compared to PN40024, where the locus spans for a length of 8.5 Mb in chromosome 3, in Mgaloblishvili, the *Rpv30* locus spans 9.2 and 9.8 Mb in haplotypes 1 and 2, respectively (Fig. 1; Table S3). Similarly, the *Rpv29* and *Rpv31* loci showed a similar trend, spanning a wider region of the Mgaloblishvili genome compared to what is observed in PN40024 (Fig. 1). In the context of the Mgaloblishvili genome, the loci appeared to extend more in haplotype 1 compared to haplotype 2. Specifically, *Rpv29* haplotype 1 covers 139.0 Kb, while *Rpv29* haplotype 2 covers 129.5 Kb. Similarly, *Rpv31* haplotype 1 spans 206.7 Kb, whereas *Rpv31* haplotype 2 covers 199.5 Kb (Fig. 1, Table S3).

### Detection of recombination events and identification of *Rpv29*, *Rpv30*, and *Rpv31* resistance haplotypes

Previous phenotype data collected from a self-fertilized progeny of Mgaloblishvili revealed a ratio of resistant and susceptible accessions suggesting a heterozygous state of the variety’s resistance loci. To further define the boundaries of the loci, particularly *Rpv30*, an analysis was conducted to detect possible recombination events in a population obtained through self-pollination of Mgaloblishvili. Additionally, the collected data were used to identify the allelic forms associated with resistance. To achieve this, the genomes of four accessions from the Mgaloblishvili self-pollinated population were sequenced using short-read sequencing technology. For accessions 97 and 145 LIB, a 39X coverage was achieved, while for accession 124 and 157 M, coverage values of 52X and 55X were obtained, respectively (Table S4). The sequencing data were used to detect recombination events in the resistant progeny and to identify resistant haplotypes. The analysed accessions were selected based on phenotype data from Sargolzaei *et al*. [14]. Among the selected accessions, two (97, 124) showed inherited resistant traits, while the other two accessions (145LIB, 157 M) were susceptible to *P. viticola*.

Different recombination events were detected on all the chromosomes of the three resistance loci (Figs S1 and S2). Short-read sequencing data of resistant accession 97 revealed the highest number of recombination events, affecting all the relevant chromosomes. On chromosome 03, a recombination event was found 2.5 Mb upstream of *Rpv30* (Fig. S1). For chromosome 14, two recombination events were detected, one 16.0 Mb upstream of *Rpv29*, and the other 7.0 Mb downstream of the locus (Fig. S1). Lastly, on chromosome 16, a single recombination event was found 5.0 Mb upstream of *Rpv31* (Fig. S1). Genomic data from the resistant accession 124 revealed a recombination event on chromosome 03, 700 Kb upstream of *Rpv30* (Fig. S2) and two events on chromosome 16, one 5.0 Mb and the other 3.5 Mb upstream of *Rpv31* (Fig. S2). Unfortunately, none of all the detected recombination events were located within the regions of the target loci. For this reason, it was not possible to refine the target loci boundaries defined by the *Vitis*18kSNP genotyping array data.

**Figure 2:**
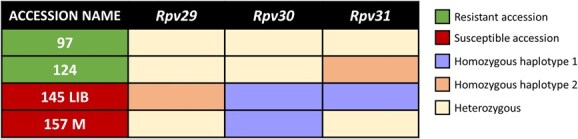
Heat map reporting the results of the genotyping for the identification of *Rpv29*, *Rpv30*, and *Rpv31* resistance haplotypes. Analysed accessions were grouped in resistant (97, 124 - green coloured) and susceptible (145 LIB, 157 M - red coloured), according to phenotype data from Sargolzaei *et al.* [14]. Each column indicates results for a single analysed locus. Resulting genotypes are shown as different colours, as indicated in the legend.

In addition to searching for recombination events, the DNA sequencing data allowed us to identify the Mgaloblishvili haplotypes segregating with resistance (Figs S3–S6). Figure 2 displays a heat map summarizing the genotyping results. *Rpv29* was found either in a heterozygous allelic state or in combination with other loci, preventing the acquisition of definitive information. Therefore, no meaningful information could be collected for *Rpv29*. Thanks to the susceptible 157 M accession, where *Rpv29* and *Rpv31* were present in heterozygous form and *Rpv30* in homozygous form for Mgaloblishvili haplotype 1, we could associate the resistance phenotype with haplotype 2 of *Rpv30*. Similarly, the resistant form of *Rpv31* was associated with haplotype 2 thanks to the resistant 124 accession. In this accession, both *Rpv29* and *Rpv30* were present in heterozygous allelic form, while *Rpv31* presented homozygous allelic form for Mgaloblishvili haplotype 2. The heterozygosity of *Rpv31* in the resistant accession 97, as well as in the susceptible accession 157 M, can be explained based on the available genomics and phenotypic data. In the resistant accession 97, all the other loci are present with a heterozygous genotype. Based on that, the presence of one copy of the resistance allele in all the loci is enough to determine the resistant phenotype. In the case of susceptible accession 157 M, the presence of heterozygosity at *Rpv31* can be explained by the presence of the susceptible haplotype in homozygosity for *Rpv30*. Nevertheless, for this locus, the result is less definitive, as there is currently no certain information available regarding the individual locus effect on the resistant phenotype [14].

### Genome comparison between PN40024 v5 and Mgaloblishvili haplotypes

**Figure 3:**
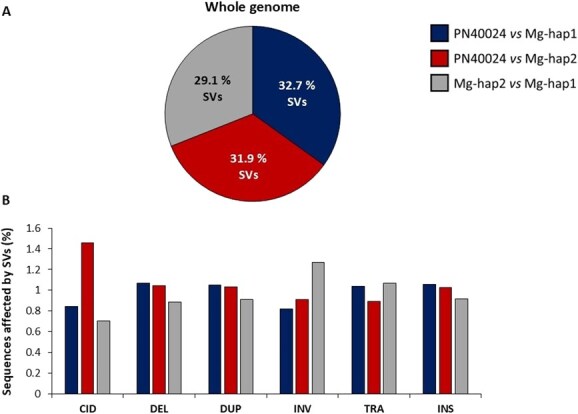
Plots representing the percentage of whole-genome sequences affected by SVs. Per each plot, PN40024 v5 genome and Mgaloblishvili haplotypes were compared. The pie chart indicates the total percentage of SVs among the three different genome comparisons (PN40024v5 *vs* Mgaloblishvili haplotype 1, PN40024v5 *vs* Mgaloblishvili haplotype 2, Mgaloblishvili haplotype 2 *vs* Mgaloblishvili haplotype 1). Bar plot shows the normalized percentage of whole-genome sequences affected by the different typologies of SVs (CID = complex insertion–deletions, DEL = deletions, DUP = duplications, INV = inversions, TRA = translocations, INS = insertions).

**Figure 4:**
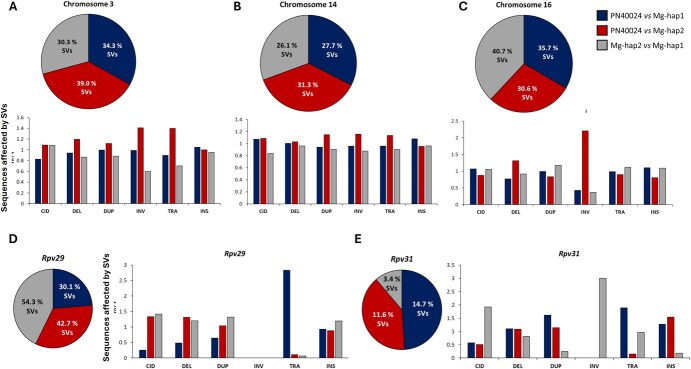
Plots reporting the percentage of sequences affected by SVs into chromosomes 3, 14, and 16 (**A–C**) and *Rpv29* and *Rpv31* resistance loci (**D–E**). Per each plot, PN40024 v5 genome and Mgaloblishvili haplotypes were compared. The pie chart indicates the total percentage of SVs among the three different comparisons (PN40024v5 *vs* Mgaloblishvili haplotype 1, PN40024v5 *vs* Mgaloblishvili haplotype 2, Mgaloblishvili haplotype 2 *vs* Mgaloblishvili haplotype 1). Bar plot shows the normalized percentage of chromosome (**A–C**) and resistance locus (**D–E**) sequences affected by the different typologies of SVs (CID = complex insertion–deletions, DEL = deletions, DUP = duplications, INV = inversions, TRA = translocations, INS = insertions).

Both haplotypes of Mgaloblishvili were aligned with PN40024 v4 and PN40024 v5 to identify SNPs and SVs (>2 bp) differentiating the three resistance loci with their respective PN40024 loci. As expected, overall there were fewer SVs between the haplotypes of Mgaloblishvili than between Mgaloblishvili and PN40024 (Fig. 3). The trend was different for the target chromosomes, as shown in Fig. 4A–C and Table 2. The percentage of SVs affecting the chromosomes ranged from 26% (chromosome 14) to 40% (chromosome 16), with duplications being the most common type of SVs. As in the case of whole-genome comparison, in chromosomes 3 and 14, fewer variants were detected between the haplotypes of Mgaloblishvili than between Mgaloblishvili and PN40024 (Fig. 4A and B). However, in the case of chromosome 16, the reverse trend was observed.

**Table 2:** Summary of the SVs identified on chromosomes (Chr) 3, 14, and 16 where *Rpv29*, *Rpv30*, and *Rpv31* resistance loci are located. SVs represents differences in the sequences of Mgaloblishvili haplotypes (Mg-hap1 and Mg-hap2) aligned to grapevine PN40024 v5 reference genome. The alignment pairs are specified in the first column. Results are indicated as percentage of SVs between the compared chromosome sequences (% of SVs). SVs evaluated where: CID = complex insertion–deletion, DEL = deletion, DUP = duplication, INV = inversion, TRA = translocation, INS = insertion. The total percentages of chromosome sequences affected by SVs is reported in the last column (Total)

**Chr3**	**Percentage (%) of SVs**						
**COMPARISON**	**CID**	**DEL**	**DUP**	**INV**	**TRA**	**INS**	**Total**
**PN40024 vs Mg-hap1**	0.2	0.5	24.4	0.2	3.0	6.0	34.3
**PN40024 vs Mg-hap2**	0.3	0.6	27.4	0.3	4.7	5.7	39.0
**Mg-hap2 vs Mg-hap1**	0.3	0.4	21.6	0.1	2.4	5.4	30.3
**Chr14**	**Percentage (%) of SVs**						
**COMPARISON**	**CID**	**DEL**	**DUP**	**INV**	**TRA**	**INS**	**Total**
**PN40024 vs Mg-hap1**	0.3	0.6	18.5	0.1	1.8	6.4	27.7
**PN40024 vs Mg-hap2**	0.3	0.6	22.5	0.1	2.2	5.7	31.3
**Mg-hap2 vs Mg-hap1**	0.2	0.6	17.8	0.1	1.7	5.7	26.1
**Chr16**	**Percentage (%) of SVs**						
**COMPARISON**	**CID**	**DEL**	**DUP**	**INV**	**TRA**	**INS**	**Total**
**PN40024 vs Mg-hap1**	0.4	0.4	24.8	0.1	3.6	6.4	35.7
**PN40024 vs Mg-hap2**	0.3	0.7	20.9	0.7	3.3	4.7	30.6
**Mg-hap2 vs Mg-hap1**	0.4	0.5	29.3	0.1	4.1	6.4	40.7

For *Rpv29*, Mgaloblishvili haplotype 1 exhibited a reduced number of structural differences when compared to the same region in PN40024 v5, in contrast to its comparison with Mgaloblishvili haplotype 2. Moreover, Mgaloblishvili haplotype 1 SVs showed a tendency to be shorter than those in Mgaloblishvili haplotype 2, when compared with PN40024 v5 (Fig. 4D). Around 50% of the locus was found to be affected by SVs (Fig. 4D, Table S5), a value that is twice the chromosome-level value (Fig. 4B, Table 2).

Concerning the *Rpv31* locus, a consistent pattern in the count of SVs was observed between PN40024 v5 and Mgaloblishvili haplotypes, mirroring the trend observed in the whole-genome comparison. Specifically, fewer SVs were detected between Mgaloblishvili haplotypes than between Mgaloblishvili haplotypes and PN40024 v5 (Fig. 4E). In this case, ~20% of the locus exhibited SVs, representing half the proportion observed at the chromosome level (Fig. 4C, Table 2, Table S5). Notably, the majority of SVs within this locus affected genes (Table S6).

Due to the impossibility of adequately define *Rpv30* locus region, SVs, and SNPs data, as well as gene content as described in the next section, were not analysed for the locus.

### *Rpv29* and *Rpv31* gene content

**Figure 5:**
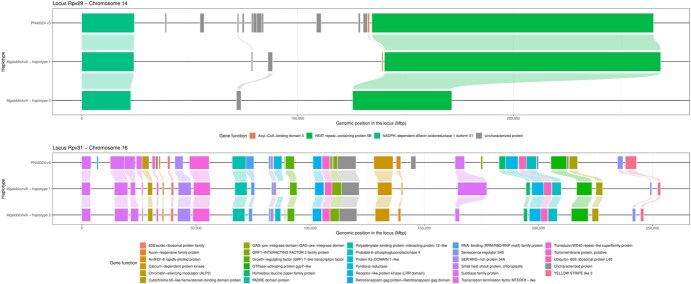
Structural representation of *Rpv29* and *Rpv31* resistance loci. Per each locus, gene content in grapevine PN40024 v5 genome and Mgaloblishvili haplotypes are shown. Each gene is indicated by genome ID and represented by a different colour. Uncharacterized proteins are shown in grey. Gene functions are also reported. Genomic position is indicated in base pairs (bp).

Gene content in the resistance loci was analysed by comparing Mgaloblishvili haplotypes to the corresponding regions in PN40024 v5. For this reason, gene annotation was validated using GMAP to map each haplotype gene content against the other. Only protein-coding genes and those validated by RNA-seq data were considered present within the target loci. Genes only partially mapping or not supported by transcriptomics data were reported in a separate file as supplementary information (Table S6). For the *Rpv29* locus, 13 coding genes were identified into PN40024 v5 genome, among them a nicotinamide adenine dinucleotide phosphate oxidase (NADPH)-dependent diflavin oxidoreductase (VITVvi_vMgal_v1.0.hap1.chr14.ver1.0.g208030; VITVvi_vMgal_v1.0.hap2.chr14.ver1.0.g500550), three uncharacterized proteins, an acyl-CoA-binding protein gene (VITVvi_vMgal_v1.0.hap1.chr14.ver1.0.g208040), and a HEAT repeat-containing protein gene (VITVvi_vMgal_v1.0.hap1.chr14.ver1.0.g208050; VITVvi_vMgal_v1.0.hap2.chr14.ver1.1.g500560) (Fig. 5, Table S6).

The Mgaloblishvili haplotypes were found to be missing some of these genes. In particular, Mgaloblishvili *Rpv29* haplotype 2 lacked more genes (3 out of 14 genes) compared to haplotype 1 (which lacked nine genes), specifically several uncharacterized proteins and the acyl-CoA-binding protein genes present in haplotype 1 (Fig. 5). In *Rpv29*, two large SVs were identified affecting protein-coding genes. The first SV spans 1092 bp, while the second spans 659 bp, leading to the absence of the uncharacterized protein 3 and acyl-CoA-binding protein genes in Mgaloblishvili *Rpv29* haplotype 2 (Fig. 5, Table S7). Several SNPs were identified in the NADPH-dependent diflavin oxidoreductase gene and HEAT repeat-containing protein gene when comparing Mgaloblishvili haplotypes and PN40024, while only one SNP was identified in acyl-CoA-binding protein gene between PN40024 v5 and Mgaloblishvili haplotype 1. In the three genes, none of the SNPs were found to cause nonsense mutations. Along with multiple small SVs, a 1035-bp insertion was identified on Mgaloblishvili NADPH-dependent diflavin oxidoreductase gene; however, the variant was found to not affect the coding sequence. On the contrary, several large SVs (>49 bp) were identified on the HEAT repeat-containing protein gene, and two of those insertions (478 and 2410 bp) were identified as the cause of a frameshift mutation in Mgaloblishvili haplotype 2 gene (Fig. 5, Table S7). Gene expression was studied using RNA-seq data collected during infection with *P. viticola* at 24, 48, and 72 h after inoculation (hai). In general, except the acyl-CoA-binding protein gene (average expression value as Transcripts per million (TPM) = 61.7), the genes within the *Rpv29* locus were highly expressed across the different time points (TPM > 450) (Table S8). Haplotype 1 genes showed higher expression compared to haplotype 2. No significant changes in the expression of the genes were detected across time points (Table S8).

Compared to the *Rpv29* locus (14 genes/132.7 kb PN40024, 5 genes/139.0 kb Mgaloblishvili haplotype 1, 3 genes/129.5 kb Mgaloblishvili haplotype 2), the *Rpv31* locus displayed a denser gene content, in both PN40024 v5 (39 genes/194.5 kb) and Mgaloblishvili haplotypes (31 genes/206.7 kb and 32 genes/199.5 kb for haplotypes 1 and 2, respectively) (Fig. 5, Table S6). With the exception of a subtilase protein gene (VITVvi_vMgal_v1.0.hap1.chr16.ver1.1.g241361, VITVvi_vMgal_v1.0.hap2.chr16.ver1.1.g533111), a transcription termination factor MTERF8-like gene (VITVvi_vMgal_v1.0.hap1.chr16.ver1.1.g241362; VITVvi_vMgal_v1.0.hap2.chr16.ver1.1.g533112) and an LRR-receptor-like protein kinase (VITVvi_vMgal_v1.0.hap1.chr16.ver1.0.g241490; VITVvi_vMgal_v1.0.hap2.chr16.ver1.0.g533240), all the locus genes were impacted by SVs, mainly small indels and SNPs, with none of them supposedly causing nonsense mutations (Fig. 5, Table S7).

**Figure 6:**
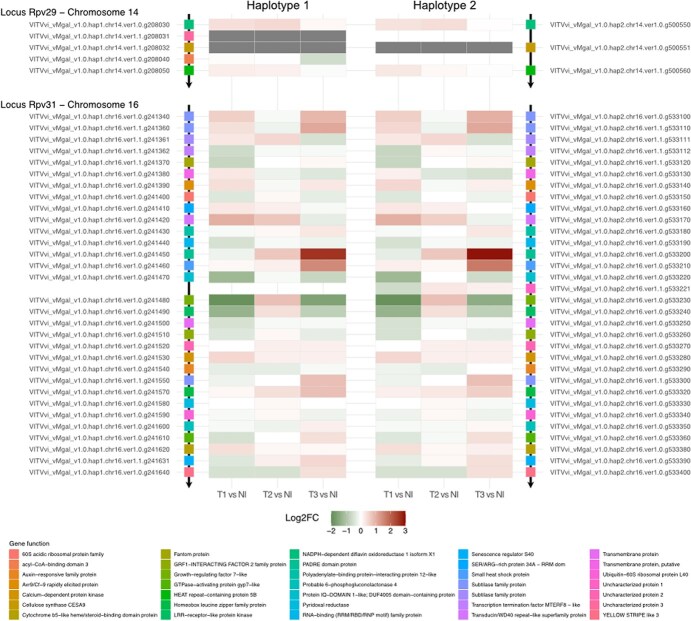
Heat maps displaying *Rpv29* and *Rpv31* gene expression during Mgaloblishvili leaf infection with *P. viticola*. Gene expression was evaluated at 24, 48, and 72 hai by RNA-seq data analysis. Genes expression values are shown as Log_2_FoldChange (Log2FC). In the heat map, downregulated genes are shown in green, while upregulated genes are shown in red. Genes are indicated by genome ID and a different colour. Gene colour scheme was maintained consistent with Fig. 4. Per each gene, predicted functions are also reported.

### *Rpv29* and *Rpv31* gene expression

The investigation of Mgaloblishvili *Rpv31* gene expression unveiled a greater degree of variability in gene regulation compared to the *Rpv29* locus (Table S8). The expression levels of *Rpv31* genes exhibited differential regulation across various time points. However, only three genes met the predefined criteria for statistical significance (*P-adjust <* .05). A gene belonging to the transducin/WD40 repeat-like superfamily protein (VITVvi_vMgal_v1.0.hap1.chr16.ver1.0.g241420; VITVvi_vMgal_v1.0.hap2.chr16.ver1.0.g533170) was upregulated in Mgaloblishvili-infected leaves at 24 hai (haplotype1-LogFC = 1.0, haplotype2-LogFC = 1.0), with its expression decreasing at later time points. The Growth-regulating factor 7-like gene (VITVvi_vMgal_v1.0.hap1.chr16.ver1.0.g241480; VITVvi_vMgal_v1.0.hap2.chr16.ver1.0.g533230) and a receptor-like protein kinase gene (VITVvi_vMgal_v1.0.hap1.chr16.ver1.0.g241490; VITVvi_vMgal_v1.0.hap2.chr16.ver1.0.g533240) were significantly downregulated in Mgaloblishvili during infection at 24 hai. The growth-regulating factor 7-like gene showed an initial downregulation equal to LogFC = −1.9 in both *Rpv31* haplotypes, followed by small upregulation at 48 hai, and a statistically significant downregulation at 72 hai (haplotype1-LogFC = −1.6; haplotype1-LogFC = −1.4). The Receptor-like protein kinase gene showcased the same pattern of expression at 48 and 72 hai, but with smaller LogFC values, lacking statistical significance (Table S8).

## Discussion

Disease-resistant grapevine cultivars could serve as an effective solution to mitigate the destructive effects of downy mildew and to shield existing fungicides from the adverse consequences of fungicide resistance, which could impair our future ability to manage the disease. Exploring new sources of resistance in *V. vinifera*, such as the *Rpv29*, *Rpv30*, and *Rpv31* loci found in Mgaloblishvili, offers significant benefits. Understanding the unique characteristics of Mgaloblishvili could yield novel resources for breeding programmes. This knowledge is especially valuable given the closer genetic relationship among these resources, thereby enhancing the efficiency of breeding efforts. In this study, we generated a chromosome-level diploid genome with a minimal amount of unplaced sequences (~11 Mb) that allowed a detailed analysis of the Mgaloblishvili downy mildew resistance loci. Data based on PN40024 were transferred to the Mgaloblishvili genome with minor compatibility issues, confirming the validity and versatility of *Vitis*18kSNP genotyping array across grapevine germplasm [22]. Comparative analysis between the PN40024 genome and Mgaloblishvili haplotypes revealed that the three loci are expanded in Mgaloblishvili (Fig. 1). Despite the increased locus size, there is no corresponding increase in gene content within the target loci (Fig. 5). In fact, Mgaloblishvili haplotypes for both *Rpv29* and *Rpv31* exhibited reduced gene content (Fig. 5).

### *Rpv29* harbours elements associated with loss of susceptibility to downy mildew

Susceptibility (S)-genes are genes essential for the interaction between plants and pathogens, critically facilitating their compatibility [23]. Based on their role in this interaction, S-genes are divided in three groups: (i) plant genes involved with the pathogen colonization, (ii) genes related to the modulation of host defences, and (iii) plant genes that allow pathogen sustenance [24]. Therefore, mutation or loss of S-genes can limit the pathogen ability to cause the disease, resulting in durable plant resistance. For this reason, despite their frequent role in plant core metabolism, S-genes could be useful resources for breeding of resistant varieties [23].

Previous studies on Mgaloblishvili identified a candidate gene for susceptibility to downy mildew, *VviLBDIf7* [25], which encodes a LOB (LATERAL ORGAN BOUNDARIES) domain-containing (LBD) protein. The transient knockdown of this gene in Pinot noir plants caused a significant reduction of susceptibility to downy mildew [13]. Notably, this study identified within the *Rpv29* locus a gene encoding a HEAT repeat-containing protein, also as a putative gene for susceptibility to downy mildew. This gene is part of the group of proteins containing HEAT repeats and is likely a member of the SWEETIE family based on its sequence. Some proteins with HEAT repeat domains are involved in non-host and basal resistance against bacterial pathogens [26]. This protein may also be involved in sugar transport and flux [27], processes known to contribute to biotic stress responses [28]. This gene exhibits several SNPs and large SVs in comparison to PN40024. Two of these variants have been identified as the cause of a frameshift mutation in the Mgaloblishvili haplotype 2 gene, significantly shortening its sequence. RNA-seq data analysis revealed that this gene is highly and constitutively expressed in Mgaloblishvili leaves, a pattern that is maintained during infection with *P. viticola*. In *Arabidopsis thaliana*, the SWEETIE gene (At1g67140) mutants are characterized by the accumulation of different sugars and an overexpression of genes involved in ethylene biosynthesis [29]. Moreover, some mutants can be maintained only as heterozygotes, due to their involvement in plant fertility [26]. Given the strong homology of this gene with *A. thaliana* SWEETIE genes, and its expression pattern, the HEAT repeat-containing protein gene may act as a grapevine S-gene. As previously reported, S-genes can serve as providers of signals that attract pathogens and increase their aggressiveness or contribute to the *in planta* accommodation of pathogens [23]. In this case, given the putative involvement of the HEAT repeat-containing protein gene in primary metabolism and hormonal signalling pathways, we can hypothesize that the pathogen relies on its functionalities for successful infection establishment. Therefore, the mutation in haplotype 2 compromises the pathogen ability to effectively manipulate the plant metabolism to its own benefit.

Although resistance conferred by loss or alteration of S-genes is generally considered recessive [23], the literature includes examples of heterozygous mutations in S-genes that were sufficient to enhance plant resistance to pathogen infections. One of these examples is MLO (mildew resistance Locus O) S-genes [30]. Wan *et al*. [30] showed that grapevine plants carrying heterozygous mutation of MLO genes showed a significant increase in plant responses associated to defence against *Erysiphe necator*. Based on available data, a similar hypothesis could be proposed for the *Rpv29* HEAT repeat-containing protein gene. This is particularly relevant considering the deleterious effects on plant fertility observed with homozygous mutations in AtSWEETIE protein genes [29].

### *Rpv31* serves as a key component of early onset response against *P. viticola*.

Mgaloblishvili defence mechanism against *P. viticola* was theorized to be based on *P. viticola* growth and sporulation limitation, determined by the overexpression of genes related to pathogen recognition, the ethylene signalling pathway, the synthesis of antimicrobial compounds and enzymes, and the development of structural barriers [10]. In this work, the data on Mgaloblishvili *Rpv31* locus suggested that the distinction between Mgaloblishvili and PN40024 may lie in the differential regulation of the same genes. The analysis of *Rpv31* locus strongly supported this hypothesis.

The transcriptomic analysis of Mgaloblishvili and Pinot noir during *P. viticola* infection indicates that the two varieties regulate similar genes. Nevertheless, Mgaloblishvili activates them within the first 24 h postinoculation, whereas Pinot noir does so after 72 h [10]. This Pinot noir delayed response correlates with reduced effectiveness in combatting the infection [10]. For *Rpv31* locus, three statistically significant differentially regulated genes were found. The upregulation of two of them in Mgaloblishvili, a transducin/WD40 repeat-like superfamily protein and a growth-regulating factor 7-like gene, is associated to biotic stress positive response and defence mechanisms to different pathogens [31, 32]. Furthermore, many genes associated with biotic stress responses were found to be constitutively highly expressed in this locus. The extensive and frequent presence of small SVs and SNPs on *Rpv31* genes, coupled with expression data, suggest the presence of the firsts as major determinants of Mgaloblishvili phenotype. Decades of studies have shown that SVs are important in plant evolution and agriculture, affecting traits such as shoot architecture, flowering time, fruit size, and stress resistance [33]. Compared to SNPs, they can cause large-scale perturbations of *cis*-regulatory regions and, therefore, are more likely to cause quantitative changes in gene expression, consequently altering phenotypes [33]. Their pivotal role in grapevine domestication, and in the definition of the specie genomic landscape has been already highlighted by multiple studies [12, 19, 34]. Regarding the detection of SVs in Mgaloblishvili, our findings are consistent with previous studies on *Vitis* genomes. Notably, the study by Zhou *et al.* [12] reported evidence of strong selection against specific SVs, such as inversions and translocations, in cultivated grapes. This purifying selection has been proposed as a distinguishing marker between wild and cultivated grapevines, suggesting a potential role in the domestication process of different varieties.

Interestingly, the two haplotypes of Mgaloblishvili exhibit an SVs landscape that supports these conclusions. The Caucasian variety appears to have a lower degree of domestication compared to PN40024, a feature reflected in the higher percentages of inversions and translocations detected between the Mgaloblishvili haplotypes and their comparison with PN40024. This lower degree of domestication could be attributed to the fact that Mgaloblishvili originates from one of the two primary centres of grapevine domestication, where cultivated varieties may retain more characteristics of their wild ancestors [8].

On chr16, the percentage of SVs between Mgaloblishvili haplotypes is higher than when the haplotypes are compared with PN40024, in particular considering duplications and insertions. This is probably based on the genetic background of the variety, a unique combination of traits in common with *V. vinifera* wine cultivars, and reminiscences of wild *sylvestris* grapes from one of grapevine’s centres of domestication [35]. In terms of disease resistance, different variants in the two haplotypes could cause quantitative changes in the gene expression, sufficient to respond to the pathogen invasion with increased reactivity. Moreover, the fact that the two haplotypes present a different SVs landscape could result in the impossibility of the pathogen to alter gene expression to the extent necessary to facilitate infection, thus indirectly causing resistance through loss of susceptibility. Considering *Rpv29* locus, this hypothesis is further supported by the variants role in determining the disruption of the acyl-CoA-binding protein gene in Mgaloblishvili haplotype 2 and the putative impairment of the HEAT repeat-containing protein gene copy on the same haplotype.

Speculating on previously published phenotypic data, and the results about the resistance haplotypes identification, it seems that homozygosity for at least one of the loci (i.e. *Rpv31*) can almost double the resistance level of the accession. However, more data are needed to support this hypothesis. Furthermore, the careful assessment of vine performances in relation to the presence of the resistant alleles should also be considered in future studies.

Available data suggest that the three loci could act synergistically in the defence mechanism against downy mildew. Unfortunately, some issues arose in defining *Rpv30* boundaries. The challenges encountered in defining them were primarily related to the characteristics of the population used to identify the loci. The available samples originated from a self-cross of the Mgaloblishvili variety, which had previously been utilized for locus identification [14]. Unfortunately, a single round of self-crossing did not produce a sufficient number of recombination events to break linkage disequilibrium within the region of interest on chromosome 3, where *Rpv30* is located. Further complicating the analysis, the probes used to map the region of interest were found to map to multiple locations, likely due to the presence of repetitive sequences in this region.

A pangenomic study revealed that the domesticated grapevine harboured less nucleotide-binding leucine-rich repeat receptor (NLR) genes compared to wild population [36]. In *Rpv29* and *Rpv31* loci, no significant enrichment of NLR genes was observed.

Nonetheless, *Rpv31* and *Rpv30* could be hypothesized to be especially involved in the initial stage responses against the pathogen, and in the fine-tuning of the downstream defence mechanisms. The presence in their regions of some receptors and genes involved in hormonal signalling and early response defences, such as antimicrobial enzymes and compounds, support the aforementioned hypothesis. On the other hand, *Rpv29* could be more involved in constitutive defence processes based on both structural and chemical barriers, eventually fine-tuned by the regulation based on the other two loci responses.

The utilization of *V. vinifera* germplasm holds significant promise for enhancing breeding programmes aimed at developing downy mildew-resistant varieties. This study elucidated the resistance mechanisms inherent in Mgaloblishvili, the first identified downy mildew *V. vinifera* resistant variety. The mechanism, based on loss of susceptibility along with an early onset response against the pathogen, based on non-host and basal resistance mechanisms, presents a viable approach for integrating resistance traits into breeding programmes. Particularly, these resources could be useful in programmes based on pyramiding with other robust *Rpv* loci characterized by delayed onset, thereby mitigating selective pressure on them. This strategic approach holds potential for developing resistant varieties with durable resistance.

## Materials and methods

### Plant material and DNA extraction

Young leaves of *V. vinifera* variety Mgaloblishvili were gathered from plants cultivated in the ampelographic collection of the Department of Agricultural and Environmental Sciences, University of Milan (Lombardy, Italia) and used to isolate high molecular weight (HMW) DNA at the laboratory of KeyGene Company (Wageningen, Netherlands). HMW DNA extraction was performed using the KeyGene proprietary uHMW protocol (version 3), based on nuclear extraction. Quality of the HMW DNA was assessed with a Nanodrop 2000 spectrophotometer (Thermo Fisher Scientific, IL, USA) and a Qubit 2.0 Fluorometer (Thermo Fisher Scientific), while the length distribution was verified using the Femto Pulse system (Agilent, CA, USA).

To detect resistant haplotypes and recombination events within the loci, DNA sequencing was carried out on two resistant accessions (97 and 124) and two susceptible accessions (157 M and 145LIB) selected from the Mgaloblishvili self-pollination progeny. DNA was extracted from young leaves using NucleoSpin Plant II Mini kit (Macherey-Nagel, Düren, Germany), according to manufacturer’s instructions. DNA concentration and quality were verified by Qubit 2.0 Fluorometer and agarose gel electrophoresis.

### Library preparation, genome sequencing, and DNA sequencing

PacBio libraries were prepared from Mgaloblishvili HMW DNA, according to the Procedure and Checklist - Preparing HiFi SMRTbell® Libraries using SMRT bell Express Template Prep Kit 2.0 (PN 101-853-100 Version 05, August 2021, PacBio, CA, USA). The DNA was fragmented to a target size of ~15–20 Kb using the Megaruptor 3 (Hologic Diagenode, NY, USA). A library was prepared and size-selected using the Blue Pippin prep (Sage Science, MA, USA) and its size distribution was confirmed using the Femto Pulse. Sequencing was performed on an 8 M ZMW Sequel IIe flow cell with adaptive loading over a 30-h data acquisition period. Raw data was processed in SMRT LINK version 10.1 with the CCS protocol under default settings to generate HiFi reads.

For Mgaloblishvili progeny accessions, genomic libraries for short-read sequencing were prepared from 1 μg of DNA extracted from young leaves, using the Kapa LTP library prep kit (Kapa Biosystems, MA, USA). The libraries were evaluated for quantity and quality with the Bioanalyzer 2100 High-Sensitivity chip (Agilent Technologies) before sequencing in 150-bp paired-end format on a HiSeq X Ten system (IDSeq, CA, USA).

### Genome assembly and annotation

The HiFi reads of Mgaloblishvili were assembled using Hifiasm v.0.16.1-r374 [17], testing 136 different combinations of assembly parameters. The final assembly was chosen based on minimal fragmentation and a high count of conserved full-length single-copy BUSCO genes across both haplotypes (parameters: a = 4, k = 41, w = 31, f = 25, r = 4, s = 0.7, D = 3, N = 300, n = 25). The draft genome underwent quality control and was scaffolded into chromosome-scale pseudomolecules using HaploSync [18] and the *Vitis* consensus genetic map from Zou *et al*. [37]. Two cycles of HaploFill were applied to enhance the completeness of the pseudomolecule assembly. Genome annotation for Mgaloblishvili followed methods outlined in previous studies [38, 39]. Functional annotations were assigned by homology with proteins in the RefSeq plant protein database (ftp://ftp.ncbi.nlm.nih.gov/refseq, retrieved 17 January 2017) and with functional domains identified through InterProScan ver. 5 [40].

### Loci identification through SNP probes and genome alignment

The *Rpv29*, *Rpv30*, and *Rpv31* loci were identified in the Mgaloblishvili genome using probes from the *Vitis*18kSNP genotyping array (Illumina Inc., CA, USA), as described in Sargolzaei *et al*. [14]. These loci regions were reconstructed based on probes that showed a positive signal in the GWAS. Locus boundaries were then established by detecting breaks in linkage disequilibrium between the signal probes and other probes on the SNP chip within the progeny of Mgaloblishvili self-pollination (data from Sargolzaei *et al*. [14]). Since the *Vitis*18kSNP genotyping array was designed on PN40024 12X v2, the probes were aligned against PN40024 v4, PN40024 v5, and Mgaloblishvili assemblies, using BLASTn-short [41], to validate their locations. For each locus, the identified regions in PN40024 v5 were then aligned to the Mgaloblishvili first haplotype, using NUCmer from MUMmer 4.0.0beta5 tool suite [42]. Subsequently, homologous regions in the first haplotype were aligned against haplotype 2 of the Mgaloblishvili genome. The gene content of each locus was characterized using Integrative Genomics Viewer (IGV) v.2.16.2 [43]. This analysis used genome annotation files along with RNA-seq alignment files, with manual curation applied to ensure annotation accuracy.

### Refinement of resistance loci location and identification of the resistance-related haplotypes

Based on the crossings data reported in Sargolzaei *et al.* [14], Mgaloblishvili was hypothesized to carry resistance in heterozygous state. To identify resistance haplotypes for the three loci and detect recombination events within these regions, deep sequencing was conducted on two resistant (97 and 124) and two susceptible (157 M and 145LIB) accessions from Mgaloblishvili’s self-pollinated progeny. Analysis of recombination events helped refine the boundaries of the target loci. Short-read DNA reads of each accession were quality-filtered and trimmed using Trimmomatic (0.36, parameters ‘ILLUMINACLIP:2:30:10 LEADING:7 TRAILING:7 SLIDINGWINDOW:10:20 MINLEN:36’). Filtered paired reads were mapped to the Mgaloblishvili diploid genome assembly, using BWA mem (unpublished). Alignments were processed with Lumpy to identify the crossing-over breakpoints and with samtools depth [44] to assess sequencing coverage and determine the zygosity of each allele at the loci. Loci were considered heterozygous if both parental haplotypes had similar coverage; otherwise, if coverage favoured one haplotype with almost null coverage on the other, they were considered homozygous. Bedtools [45] was then used to evaluate these zygosity differences across 10-kb windows within the regions of interest.

### Genome comparisons for the detection of structural variants and SNPs

To identify differences between grapevine PN40024 v5 genome and Mgaloblishvili’s haplotypes, pairwise comparisons of each haplotype pair were conducted using NUCmer from the MUMmer 4.0.0beta5 suite [42], with – mum option. Alignments were filtered and used for variant calling of both SNPs and identification of long SVs using show-snps and show-diff form MUMmer suite. Output files were converted to BED format to facilitate comparison of the detected variants. These analyses were also applied to regions within the loci of interest. Bedtools [45] was then used to compare the variant locations with gene annotations in these loci to determine which gene features were affected by the identified variants.

### RNA sequencing data analysis for loci genes expression

RNA-seq data from [10] (ENA project code: PRJEB24540) were used to analyse gene expression within the loci of interest. Data from Mgaloblishvili leaves inoculated and not inoculated with *P*. *viticola,* and collected at 24, 48, and 72 hai, were used for the purpose. After quality control and preprocessing, reads were mapped to the Mgaloblishvili diploid genome, using HISAT2 [46] with - - sensitive and - - k 50 parameters. Genome alignments were adjusted to transcriptome alignments using the genome’s gene annotation employing Ubu 1.2 (https://github.com/mozack/ubu). Transcript abundance was quantified with Salmon 1.5.2 [47], using the parameters - - seqBias and - - posBias. Quantification files were imported using an R package, tximport v.1.20.0 [48], and DESeq2 v.1.16.1 [49] was used to assess the gene-level expression and modulation within the annotated loci. Genes expression results were reported as transcripts per million (TPM) and as log fold change (logFC). Genes with a LogFC >1 and < −1 and an adjusted *P* < .05 were considered significantly differentially expressed across time points.

## Supplementary Material

Web_Material_uhaf055

## Data Availability

Sequencing data are accessible through NCBI under the BioProject ID PRJNA1087404. New genome assembly of Mgaloblishvili and annotation files are available at Zenodo under the DOI: 10.5281/zenodo.10815333 and at www.grapegenomics.com.
