# Supplementary material for: The genome of Vitis vinifera cv. Mgaloblishvili reveals resistance and susceptibility factors to downy mildew in the Rpv29 and Rpv31 loci
Source: Hortic Res. 2025 Feb 20;12(6):uhaf055. doi: 10.1093/hr/uhaf055 (PMC12017795; doi:10.1093/hr/uhaf055)
Supplement: Web_Material_uhaf055 [file web_material_uhaf055.zip › Figure S1.docx]

**
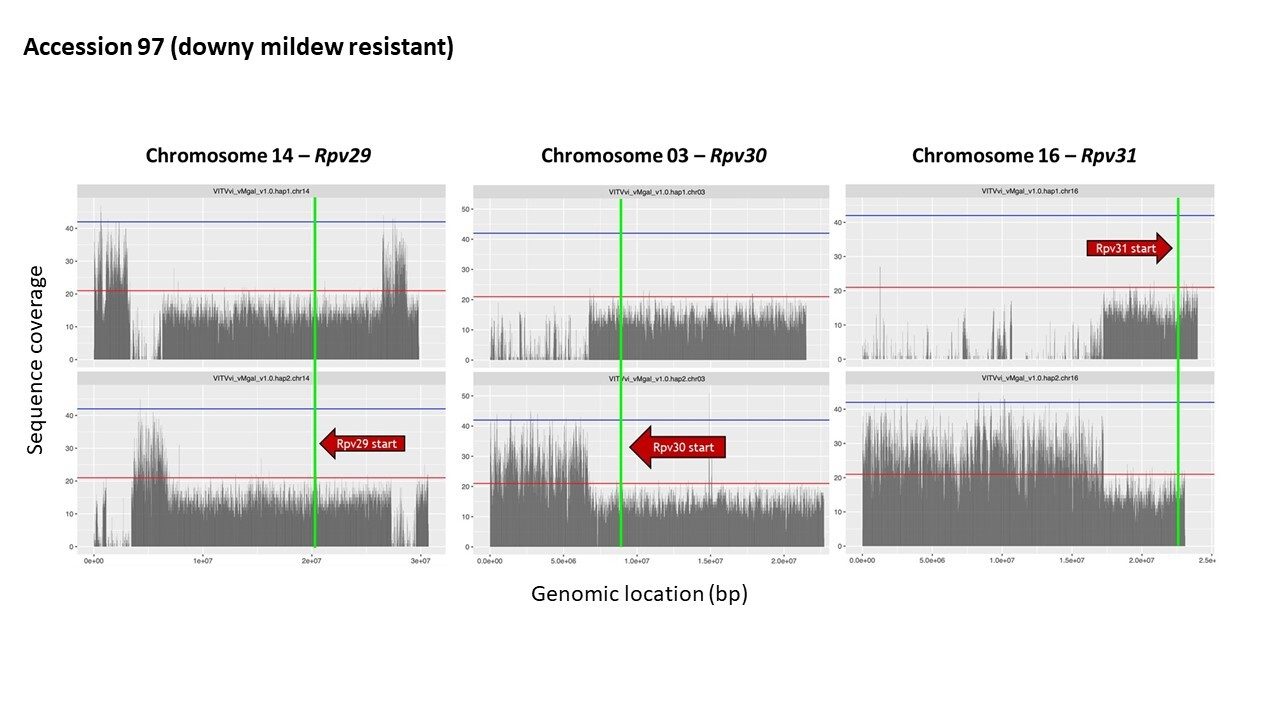
**

**Figure S1** Identification of recombination events on chromosome 14 (*Rpv29* location), 3 (*Rpv30* location) and 16 (*Rpv31* location) of accession 97 (downy mildew resistant). The accession is part of Mgaloblishvili self-pollinated progeny. DNA-seq reads alignments against the chromosomes of Mgaloblishvili genome haplotypes (parental haplotypes) are shown. Blue line represents the average distribution value of reads in homozygous allelic state for a determined parental haplotype. Red line represents the average distribution value of reads in heterozygous allelic state for the parental haplotypes. Green vertical line and red arrow indicates *Rpv* locus start position. Recombination events are represented by switches in reads coverage distribution.
