# Supplementary material for: The genome of Vitis vinifera cv. Mgaloblishvili reveals resistance and susceptibility factors to downy mildew in the Rpv29 and Rpv31 loci
Source: Hortic Res. 2025 Feb 20;12(6):uhaf055. doi: 10.1093/hr/uhaf055 (PMC12017795; doi:10.1093/hr/uhaf055)
Supplement: Web_Material_uhaf055 [file web_material_uhaf055.zip › Figure S2.docx]

**
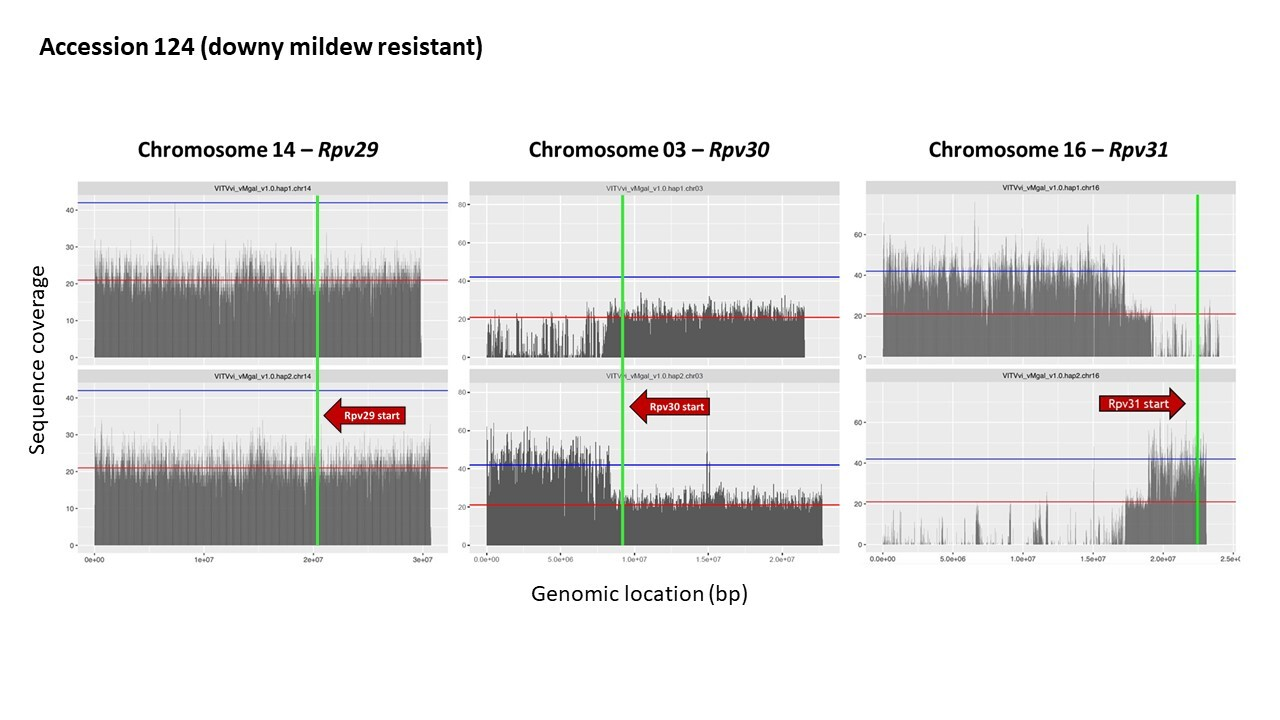
**

**Figure S2** Identification of recombination events on chromosome 14 (*Rpv29* location), 3 (*Rpv30* location) and 16 (*Rpv31* location) of accession 124 (downy mildew resistant). The accession is part of Mgaloblishvili self-pollinated progeny. DNA-seq reads alignments against the chromosomes of Mgaloblishvili genome haplotypes (parental haplotypes) are shown. Blue horizontal line represents the average distribution value of reads in homozygous allelic state for a determined parental haplotype. Red horizontal line represents the average distribution value of reads in heterozygous allelic state for the parental haplotypes. Green vertical line indicates putative recombination event. Green vertical line and red arrow indicates *Rpv* locus start position. Recombination events are represented by switches in reads coverage distribution.
