# Supplementary material for: The genome of Vitis vinifera cv. Mgaloblishvili reveals resistance and susceptibility factors to downy mildew in the Rpv29 and Rpv31 loci
Source: Hortic Res. 2025 Feb 20;12(6):uhaf055. doi: 10.1093/hr/uhaf055 (PMC12017795; doi:10.1093/hr/uhaf055)
Supplement: Web_Material_uhaf055 [file web_material_uhaf055.zip › Figure S6.docx]

**
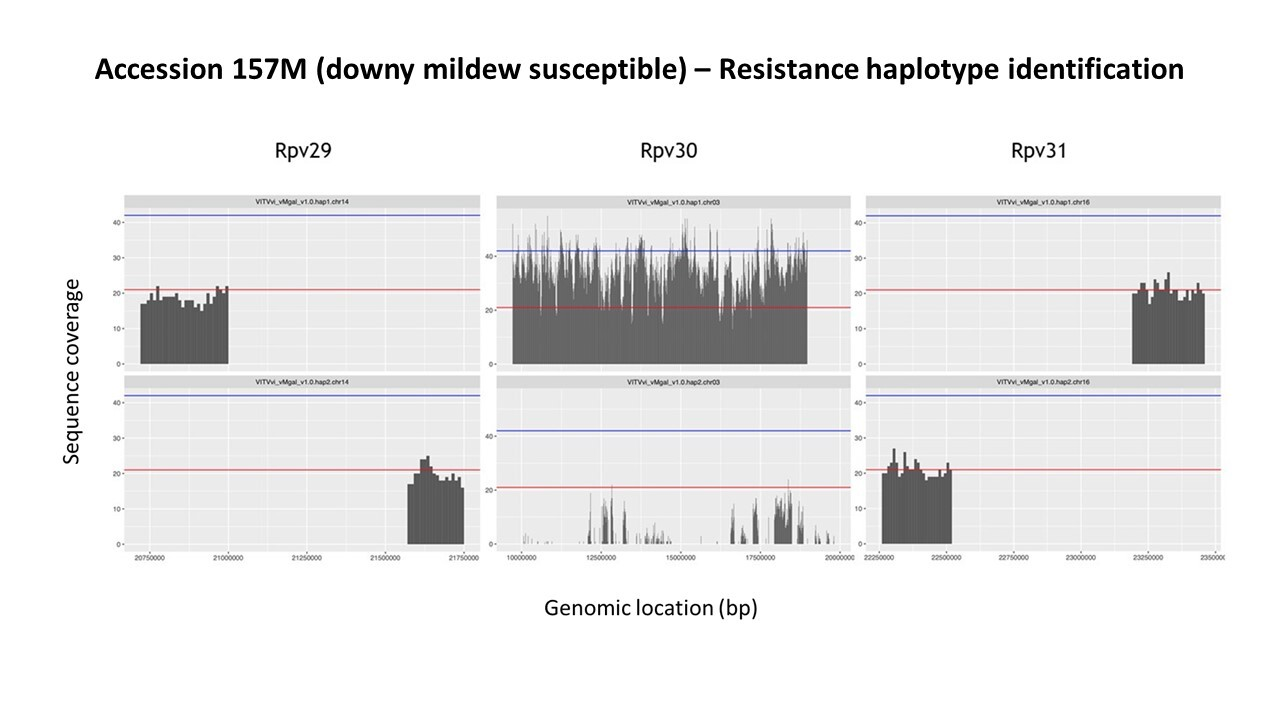
**

**Figure S6** Analysis of *Rpv29* (chr14), *Rpv30* (chr3) and *Rpv31* (chr16) allelic state in accession 157M (downy mildew susceptible). The accession is part of Mgaloblishvili self-pollinated progeny and was analysed to determine the resistance haplotype of the target loci. DNA-seq reads alignments against the three loci regions in Mgaloblishvili genome haplotypes (parental haplotypes) are shown. Blue horizontal line represents the average distribution value of reads in homozygous allelic state for a determined parental haplotype. Red horizontal line represents the average distribution value of reads in heterozygous allelic state for the parental haplotypes.
